# Obesity Disease and Surgery

**DOI:** 10.1155/2014/652341

**Published:** 2014-04-28

**Authors:** Abdulrahman Saleh Al-Mulhim, Hessah Abdulaziz Al-Hussaini, Bashaeer Abdullah Al-Jalal, Rehab Omar Al-Moagal, Sara Abdullah Al-Najjar

**Affiliations:** ^1^Medical College, King Faisal University, Saudi Arabia; ^2^Department of Surgery, Medical College, King Faisal University, Hofuf, P.O. Box 1164, Al-Hassa 31982, Saudi Arabia

## Abstract

Obesity is a medical disease that is increasing significantly nowadays. Worldwide obesity prevalence doubled since 1980. Obese patients are at great risk for complications with physical and psychological burdens, thus affecting their quality of life. Obesity is well known to have higher risk for cardiovascular diseases, diabetes mellitus, musculoskeletal diseases and shorter life expectancy. In addition, obesity has a great impact on surgical diseases, and elective surgeries in comparison to general population. There is higher risk for wound infection, longer operative time, poorer outcome, and others. The higher the BMI (body mass index), the higher the risk for these complications. This literature review illustrates the prevalence of obesity as a diseases and complications of obesity in general as well as, in a surgical point of view, general surgery perioperative risks and complications among obese patients. It will review the evidence-based updates in these headlines.

## 1. Introduction

Obesity is a medical disease that is increasing significantly nowadays. It is classically defined as a body mass index ≥30 kg/m^2^. Almost all healthcare professionals are exposed to obese patients because of their higher risk for morbidity and mortality, and surgeons are not exceptions [1].

Obesity and its severity can be measured by several methods [2]. They include the following.

- Body mass index (BMI) [1]: this is the most common method to measure obesity in adults (see Table 1(a)) and children (see Table 1(b)).
- Skin fold thickness [2] (biceps, triceps, subscapular, and suprailiac): it measures the subcutaneous fat to determine the percentage of body fat.
- Waist circumference [2]: it is a common method to measure the risk of cardiometabolic affection (see Table 2).
- Waist-to-hip ratio [2]: it examines fat distribution and it is used less frequently.
- Waist-to-height ratio [3]: waist-to-height ratio is a better screening tool than waist circumference and BMI for adult cardiometabolic risk factors in both sexes (see Table 3).

## 2. Prevalence of Obesity

### 2.1. Worldwide

Worldwide obesity prevalence doubled since 1980 [4]. In 2010, approximately 1.0 billion adults are overweight, and a further 475 million are obese [5]. It was also estimated in 2010 that up to 200 million school-aged children are either overweight or obese; of those, 40–50 million are classified as obese [5, 6].

### 2.2. Regional (Saudi Arabia)

In 2005, the estimated overall obesity prevalence in Saudi Arabia was 35.5% [7]. In 2010, Saudi Arabia was ranked at the 11th place for obesity worldwide, with obese men making 26.4% and obese women making 44% among general Saudi population [8]. From a collected data in 2004 and 2005 from Saudi children, the overall prevalence of overweight was 21% and 13.4% and obesity was 9.3% [9].

## 3. Risk of General Diseases Associated with Obesity

### 3.1. Mortality

Overweight and obesity are the fifth leading risk for global deaths [4]. It was thought that mortality rates from noncardiovascular diseases were inversely related to BMI [10]. But now obesity is highly associated with overall mortality among adults [11–14] (as well as in children [15]). The higher the BMI, the higher the risk. The high mortality rate is mainly due to vascular diseases [16] and cancers [17]. In addition, weight fluctuation is associated with higher risk of overall mortality [18].

### 3.2. Diabetes Mellitus

Obesity is highly linked to the development of type 2 diabetes mellitus at all ages [19–21]. Increase in the BMI [20] and waist circumference [21] increases the risk of type 2 diabetes mellitus; this is because of the common association between obesity and metabolic syndrome, impaired fasting glucose, and impaired glucose tolerance [22].

### 3.3. Hypertension

As the prevalence of obesity increases, the prevalence of arterial hypertension increases as well [23, 24]. Generalized [23, 24] and central obesity [25] increase the risk of arterial hypertension. Since high blood pressure and impaired glucose tolerance are frequently associated with obesity, it has been suggested that hyperinsulinemia could represent one of the pathogenic connection between obesity and arterial hypertension [26]. Age, race, and sex may alter the frequency of hypertension in obese patients [27].

### 3.4. Dyslipidemia

Obesity has strong association with atherogenic dyslipidemia, characterized by high triglycerides and low high-density lipoprotein (HDL) cholesterol [28], while central obesity is associated with a triad of high triglycerides, low HDL cholesterol, and high low-density lipoprotein (LDL) cholesterol [29].

### 3.5. Heart Diseases

Obesity is considered a risk factor for multiple heart diseases like coronary artery disease, heart failure, and atrial fibrillation. Despite the old debate to consider obesity as a risk factor of coronary artery disease [30], this relation is confirmed by higher BMI [31] and higher waist circumference [32], but waist-to-hip ratio can replace the BMI and waist circumference in being a better predictor of coronary artery disease [33]. It is more prevalent in females as compared to males [34]. Surprisingly, obesity is associated with more favorable short-term outcomes after acute coronary syndrome [35]. Obesity has a strange association with heart failure that is called obesity paradox (as well as other diseases). It can result in systolic and diastolic dysfunction [36]; on the other hand, obese patients with heart failure have better clinical outcomes in comparison to patients with normal BMI [37, 38]. Obesity (regarding high BMI and waist circumference) has also higher risk for atrial fibrillation incidence, recurrence, and poor prognosis [39]. Obstructive sleep apnea [40] and pericardial fat [41, 42] that are commonly associated with obesity are considered significant underlying mechanisms for heart failure and atrial fibrillation.

### 3.6. Central Nervous System Diseases

Obesity has a negative impact on vascular (e.g., stroke) [43] and nonvascular diseases (e.g., Alzheimer's disease) [44]. In 2009, BMI was thought to be not significantly associated with carotid atherosclerosis [45], but it is now considered as the greatest risk factor for intima-media thickening of carotid artery and therefore predisposing to ischemic stroke [46]. BMI was a risk factor for total and ischemic stroke in men and women; however, abdominal adiposity was a risk factor for total and ischemic stroke only in men [47] (hemorrhagic stroke is not related to obesity [48]). In addition, obesity might result in executive and cognitive dysfunction, with other associated risk factors [43, 49].

### 3.7. Respiratory Diseases

Obesity is a risk factor for several respiratory diseases like obstructive sleep apnea [50] and bronchial asthma [51]. Obesity plays a major role in the pathogenesis of obstructive sleep apnea (high BMI, visceral adiposity, insulin resistance, central neural mechanisms, neck circumference, etc.) [52, 53], its severity [54], and negatively affecting in the quality of life [55]. Obesity also has a significant impact on bronchial asthma risk, severity, and control [51]. It is a risk factor for airway hyperresponsiveness [56] but not airway inflammation [57].

### 3.8. Gastrointestinal Diseases

Obesity is associated with an increased risk of esophageal disorders such as gastroesophageal reflux disease, Barrett esophagus, and esophageal adenocarcinoma [58]. Severity and duration of reflux symptoms [59] as well as Barrett esophagus [60] are related to high BMI, high waist circumference, and high waist-to-hip ratio, and this relation was not found before [61]. The risk of nonalcoholic fatty liver disease is also increased with high BMI [62] and high waist circumference [63].

### 3.9. Kidney Diseases

Obesity is related to a variety of kidney diseases, including glomerulomegaly, focal segmental glomerulosclerosis, and chronic kidney disease [64]. It is found that obesity is highly prevalent among renal transplant recipients [65]. As with obesity-survival paradox, it is associated with improved survival in hemodialysis patients [66].

### 3.10. Osteoarthritis

Obesity is a primary risk factor for the development [67] and progression [68] of knee, hip (recently involved [69]), and hand osteoarthritis. This is due to the biomechanical and metabolic effects of obesity on joints [67].

### 3.11. Gynecological and Obstetric Complications

Overweight and obesity increase the overall risk of pregnancy, childbirth, and neonatal complications [70]. Pregnancy complications include anovulation, abortion, gestational diabetes, preeclampsia [70], and gestational hypertension [71]. Delivery complications include longer first stage of labor and higher risk for cesarean section [72]. Neonatal and child complications include congenital anomalies, abnormal intrauterine growth [73], macrosomia, birth injuries, perinatal asphyxia, neonatal respiratory distress [74], and childhood obesity [73]. In addition, obesity is highly associated with polycystic ovary syndrome and increases some of its features like hyperandrogenism, hirsutism, and infertility [75].

### 3.12. Malignancies

Obesity is associated with higher cancer incidence, recurrence, progression, and death [76]. It can be responsible for the following cancer types [77]:

- gastrointestinal system [78],
- hepatobiliary system [79],
- breast (premenopausal [80] and postmenopausal),
- endometrial, ovary, and cervix [81],
- lung (but not in current or former smokers [82]),
- skin (malignant melanoma) [83],
- multiple myeloma [83],
- leukemia [83].

The increased risk of cancer mortality associated with an elevated BMI is significant at levels above 30 kg/m^2^ [84].

### 3.13. Psychological Disorders

Obesity contributes to a variety of psychological disorders in adults and children. These include depression, eating disorders, suicidal attempts, anxiety, somatization, obsessive-compulsive disorder, and others [85–90].

## 4. Surgical Diseases Associated with Obesity

### 4.1. Peptic Ulcer

Peptic ulcer is one of the complications seen in obesity, and men are more susceptible than women to develop peptic ulcer [91]. Nevertheless, it was thought that* Helicobacter pylori* infection does not increase in overweight patients [92], and peptic ulcer was inversely related to BMI [93].

### 4.2. Pancreatic Diseases

Obesity has been considered as a risk factor for pancreatic diseases, including pancreatitis and pancreatic cancer. Severe acute pancreatitis is significantly more frequent in obese patients. Furthermore, obese patients develop systemic and local complications of acute pancreatitis more frequently. Obesity is a poor prognostic factor in acute pancreatitis and overweight before disease onset appears to be a risk factor for chronic pancreatitis. Overweight and/or obesity are associated with greater risk of pancreatic cancer and younger age of onset. Obesity is associated with negative prognostic factor and increased mortality in pancreatic cancer. However, there are controversies regarding the effects of obesity on long-term postoperative results in the patient with pancreatic cancer [94].

### 4.3. Gall Bladder Diseases

In the biliary diseases, obesity and overweight have been known as major risk factors for gallstones. Obesity, insulin resistance, hyperinsulinemia, and metabolic syndrome are related to various gallbladder diseases including gallbladder stones, cholecystitis, gallbladder polyps, and gallbladder cancers [95].

### 4.4. Appendicitis

Obesity does not show any delay in the diagnosis of appendicitis [96], except in children [97]. Obese adults and children have higher risk for complications from appendicitis like perforation [97, 98].

### 4.5. Diverticulitis

BMI, waist circumference, and waist-to-hip ratio significantly increase the risks of diverticulitis, diverticular bleeding [99], perforation [100], and recurrence [101]. A previous study on few obese patients resulted in the presence of diverticulosis without diverticulitis [102].

### 4.6. Hernia

There is an association between obesity and the presence of hiatal hernia [103]. In addition, incisional hernia (although it is a postoperative complication) is highly prevalent among obese patients [104].

### 4.7. Abdominal Trauma

Obese patients are prone to vehicle accidents more than general population due to the associated sleep apnea. They usually suffer from chest, pelvis, and extremity fractures [105]. Unfortunately, they carry a high risk for morbidity and mortality because of difficult assessment and treatment [106].

## 5. Perioperative Complications Related to Obesity

### 5.1. Preoperative Complications

Obesity is an independent risk factor for perioperative morbidity, and morbid obesity is a risk factor for mortality [107]. Obesity-related comorbidities including obstructive sleep apnea place patients undergoing bariatric surgery at increased risk for complications perioperatively [108]. Problems in the perioperative management of obese patients are mainly related to their respiratory system. These can manifest as reduced lung volume with increased atelectasis; derangements in respiratory system, lung and chest wall compliance and increased resistance; and moderate to severe hypoxaemia. These physiological alterations are more marked in obese patients with hypercapnic syndrome or obstructive sleep apnea syndrome [109].

### 5.2. Intraoperative Complications

The degree of obesity influences the incidence of intraoperative surgical complications [110]. Intraoperative considerations include requirements for special equipment, patient positioning, intravenous line placement, central monitoring lines, and anesthesia specific to the physiologic changes in obese patients [111]. Also, airway management, intravenous fluid administration, physiologic responses to pneumoperitoneum during laparoscopic procedures, and the risk of thrombotic complications and peripheral nerve injuries in extremely obese patients are among the factors that present special intraoperative challenges that affect postoperative recovery of the bariatric patient [112]. In surgical regional anesthesia, obesity is associated with higher block failure and complication rates [113]. Obese and overweight patients undergoing resection for colorectal carcinoma when compared with normal-weight patients have similar intraoperative blood loss and postoperative complications but longer operative times [114].

### 5.3. Postoperative Complications

Obese patients have a significantly higher risk of postoperative myocardial infarction, wound infection, nerve injury, urinary infection [107], and DVT than do nonobese patients, and they may differ from other patients in supplemental oxygen requirements, medication dosing, and outcomes in intensive care units [115]. Obese population has a higher than normal incidence of perioperative pulmonary embolism [116]. Skin/wound problems which are common, yet more difficult to manage for these patients, include pressure ulcers, tracheostomy care (potentially resulting from ventilatory insufficiency), candidiasis, tape-related skin tears, incontinence, and lymphedema [117].

### 5.4. Laparoscopic versus Open Abdominal Surgeries

Evidence supports that laparoscopic approach results in better outcomes than open surgery with few exceptions. Laparoscopic surgery may be a safer treatment than open surgery for patients requiring bariatric surgery [118]. Laparoscopic procedures are associated with shorter operative times, less blood loss, less postoperative pain and analgesic consumption, earlier postoperative recovery, shorter hospital stays, lesser degree of abdominal wall trauma, reduction in the rate of incisional hernia and pulmonary embolism, and better respiratory function and cosmetic results [119–126]. However, some laparoscopic procedures as laparoscopic-converted colon resection are associated with significantly greater morbidity, particularly wound complications and greater length of hospital stay, compared to open surgery [127]. In some surgeries, for example, appendectomy laparoscopic technique did not have superiority over the open one for obese patients [128].

## 6. Bariatric Surgeries

Bariatric surgeries make tremendous improvement in obese patients' health and life. They decrease overall obesity complications and improve the quality of life. They are indicated in patients with BMI > 40 kg/m^2^ or >35 kg/m^2^ with complications associated with obesity such as hypertension, type 2 diabetes mellitus, and obstructive sleep apnea, as well as for those not improving with medical therapy. In very high-risk patients, staged approaches may be required in which one operation (either gastrectomy or intestinal bypass) is followed by the other operation in two separate surgical procedures [1].

### 6.1. Types

Different types of bariatric surgeries were invented and developed since early 50s, through either open or laparoscopic techniques. They can be broadly categorized into restrictive operations, malabsorptive operations, and combination operations. Restrictive operations include the adjustable gastric band and the sleeve gastrectomy. Malabsorptive operations include biliopancreatic diversion. A combination of restriction and malabsorption is represented by the Roux-en-Y gastric bypass and biliopancreatic diversion-duodenal switch [1].

### 6.2. Advantages and Disadvantages

Table 4 demonstrates different types of bariatric surgeries with their main advantages and disadvantages [129].

### 6.3. Complications of Bariatric Surgeries in General

(a)  Mortality [129]: operative (30-day) mortality for bariatric surgery ranges from 0.1% to 2%. Mortality rates depend on several factors: complexity of the operation, patient comorbidities, patient body habitus, and experience of the surgeon and the center. Restrictive operations have lower mortality risk than malabsorptive operations.
(b)  Early complications [1]: the severely obese patients are at risk of developing several general complications, such as thromboembolism, pulmonary or respiratory insufficiency, hemorrhage, peritonitis, and wound infection. Laparoscopy has been instrumental in decreasing these rates.
(c) Late complications [1]: they include the following:
  - gastrointestinal obstruction,
  - marginal ulceration that may present a frequent source of abdominal pain and anemia,
  - incisional hernias which are common after open bariatric surgery and require subsequent surgical intervention,
  - device-related complications with the gastric band which include malfunction of the band, tubing, or reservoir component,
  - hypoglycemia
  - steatorrhea, diarrhea, and bacterial overgrowth that are more common with malabsorptive procedures,
  - nutritional deficiencies of micronutrients,
  - neurological complications such as peripheral neuropathy, burning feet syndrome, meralgia paresthesia, myotonic syndrome, posterolateral myelopathy, myotonic syndrome, optic neuropathy, Wernicke-Korsakoff encephalopathy, and lumbosacral plexopathy.

## Figures and Tables

**(a):** 

Underweight	Normal	Overweight	Obese (≥30 kg/m^2^)		
			Mild	Moderate	Severe or morbid
>18.5 kg/m^2^	18.5–24.9 kg/m^2^	25–29.9 kg/m^2^	30–34.9 kg/m^2^	35–39.9 kg/m^2^	≥40 kg/m^2^

**(b):** 

Overweight	Obese	Severely obese
85th–94th percentile	95th percentile or ≥30 kg/m^2^, whichever is lower	99th percentile

**Table 2:** 

	Increased risk	Substantially increased risk
Men	≥94 cm	≥102 cm
Women	≥80 cm	≥88 cm

**Table 3:** 

	Increased risk	Substantially increased risk
Men	53.6%	58.3%
Women	49.2%	54.1%

**Table 4:** 

Procedure	Advantages	Disadvantages
Vertical banded gastroplasty	(i) No intestinal anastomosis	(i) Foreign body
	(ii) No malabsorption	(ii) High long-term failure rate

Adjustable gastric banding	(i) Technically simple	(i) Foreign body
	(ii) Low morbidity	(ii) 15–30% failure rate
	(iii) Reversible	(iii) May promote maladaptive eating behaviour
	(iv) No intestinal anastomosis	
	(v) No malabsorption	

Sleeve gastrectomy	(i) Technically simple	(i) May require second-stage procedure
	(ii) Low morbidity	(ii) Unknown long-term results

Gastric bypass	(i) Sustained weight loss	(i) Intestinal anastamoses
	(ii) Dumping in sweet eaters	(ii) Loss of access to gastric remnant
	(iii) Resolution of gastroesophageal reflux disease	(iii) Mild risk for vitamin deficiencies
		(iv) Risk of marginal ulceration

Biliopancreatic diversion-duodenal switch	(i) Excellent sustained weight loss	(i) Technically demanding
	(ii) Larger portion size	(ii) Frequent bowel movement and flatulence
	(iii) Excellent malabsorption	(iii) Increased risk of vitamin and protein malnutrition
